# Supplementary material for: Impact on Isoleucine and Valine Supplementation When Decreasing Use of Medical Food in the Nutritional Management of Methylmalonic Acidemia
Source: Nutrients. 2020 Feb 13;12(2):473. doi: 10.3390/nu12020473 (PMC7071216; doi:10.3390/nu12020473)
Supplement: Supplementary file 1 [file nutrients-12-00473-s001.pdf]

Supplementary Materials:

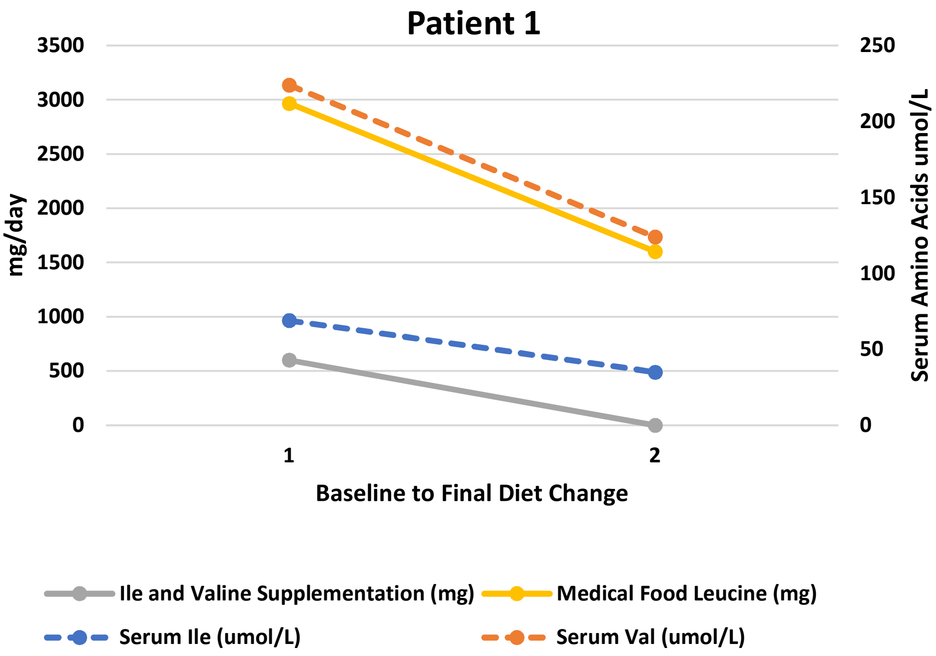

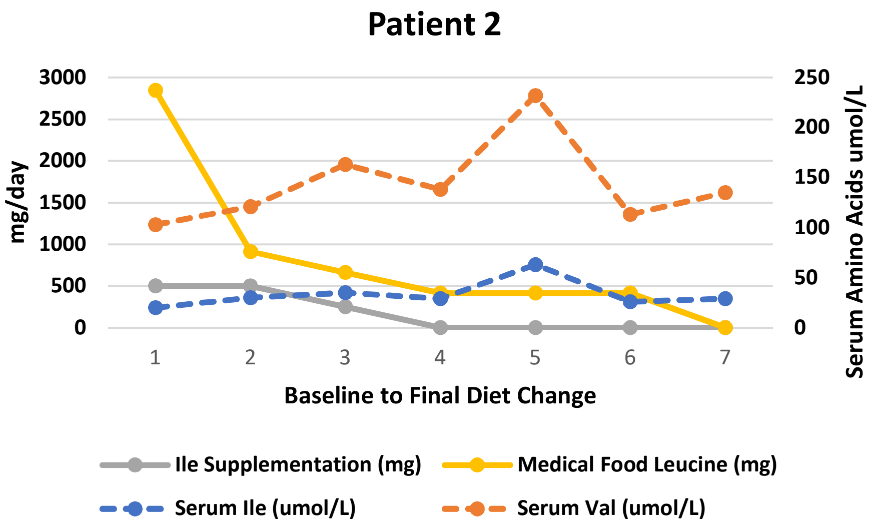

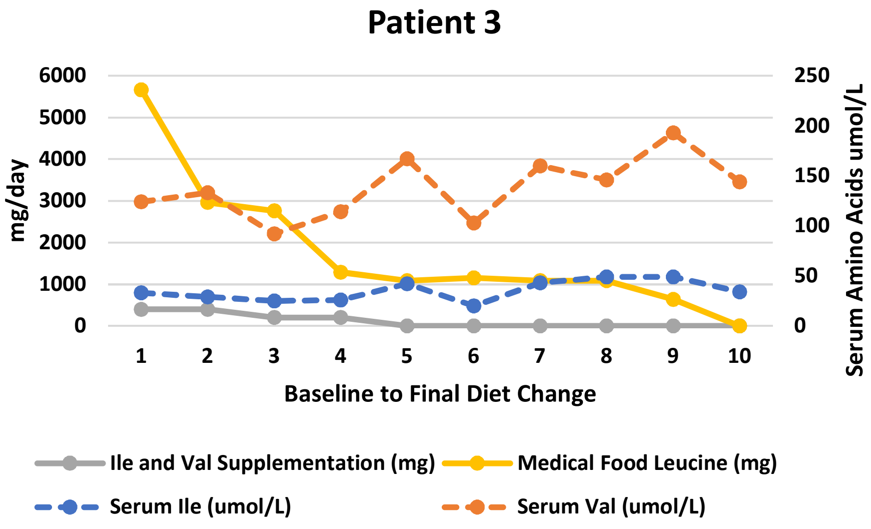
Patient 3
6000
5000
4000
3000
2000
1000
0
mg/day
250
200
150
100
50
0
Serum Amino Acids umol/L
1 2 3 4 5 6 7 8 9 10
Baseline to Final Diet Change
Ile and Val Supplementation (mg)
Medical Food Leucine (mg)
Serum Ile (umol/L)
Serum Val (umol/L)

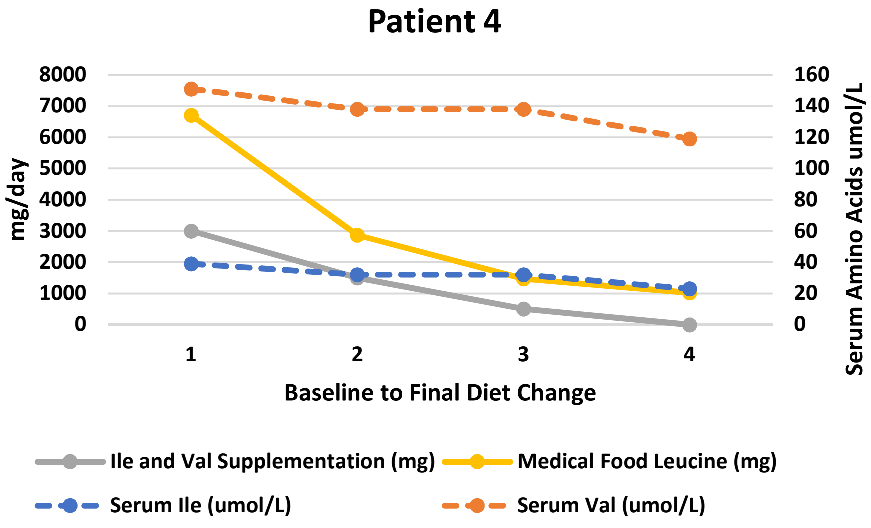
Patient 4
8000
7000
6000
5000
4000
3000
2000
1000
0
mg/day
160
140
120
100
80
60
40
20
0
Serum Amino Acids umol/L
1 2 3 4
Baseline to Final Diet Change
Ile and Val Supplementation (mg)
Medical Food Leucine (mg)
Serum Ile (umol/L)
Serum Val (umol/L)

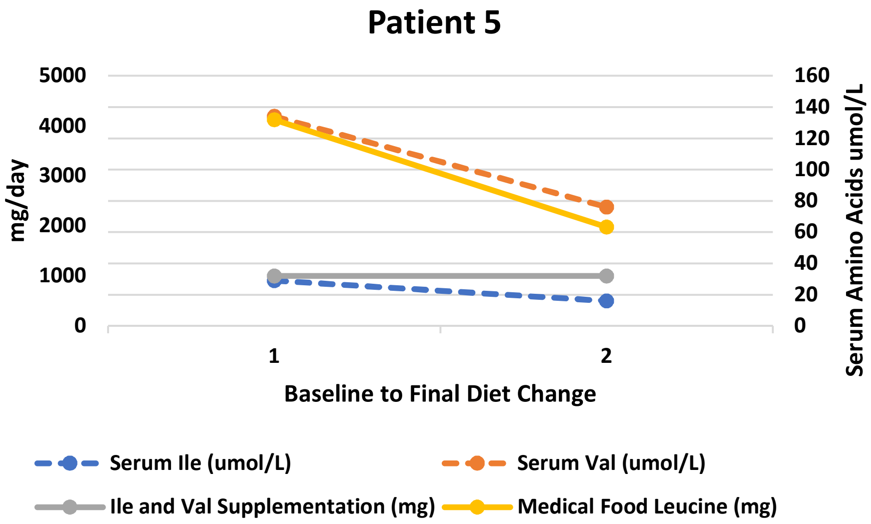

Figure S1. Individual patient results of serum amino acid levels in relation to decreased supplementation of isoleucine and valine while reducing leucine intake from precursor-free medical food.
